# A novel nonsense variant in *NSD1* gene in a female child with Sotos syndrome: A case report and literature review

**DOI:** 10.1002/brb3.3290

**Published:** 2023-10-31

**Authors:** Xinting Liu, Chen Chen, Lin Wan, Gang Zhu, Yan Zhao, Lizhu Hu, Yan Liang, Jing Gao, Jing Wang, Guang Yang

**Affiliations:** ^1^ Senior Department of Pediatrics the Seventh Medical Center of PLA General Hospital Beijing China; ^2^ Department of Pediatrics, the First Medical Center Chinese PLA General Hospital Beijing China; ^3^ Medical School of Chinese PLA Beijing China; ^4^ The Second School of Clinical Medicine Southern Medical University Guangzhou China

**Keywords:** nonsense variant, NSD1 gene, Sotos syndrome

## Abstract

**Introduction:**

Sotos syndrome (SS) is an overgrowth disease characterized by distinctive facial features, advanced bone age, macrocephaly, and developmental delay is associated with alterations in the *NSD1* gene. Here, we report a case of a 4‐year‐old female child with SS caused by *NSD1* gene nonsense mutation.

**Methods:**

Whole‐exome sequencing (WES) was applied for probands and her parents. Sanger sequencing was used to confirm the mutation. We performed the literature review using PubMed and found 12 articles and 14 patients who presented with SS.

**Results:**

The patient showed typical facial features of SS, hand deformities, and seizure. WES revealed de novo heterozygous variant: *NSD1* (NM_022455.5), c.6095G > A, p.TRP2032*. We also reviewed the phenotype spectrum of 14 patients with SS, who exhibited a variety of clinical phenotypes, including developmental delay, seizures, scoliosis, hearing loss, cardiac and urinary system abnormalities, and so on.

**Discussion:**

The lack of correlation between mutation sites or types and phenotypes was summarized by literature reviewing. The NSD1 protein contains 14 functional domains and this nonsense mutation was located in SET domain. Early appearance of the termination codon leads to protein truncation. Haploinsufficiency of the *NSD1* gene causes the overgrowth disorders.

## Graphical abstract

The nonsense mutation c.6095G>A (p.TRP2032*) in NSD1 gene associated with Sotos syndrome has not been reported before. The phenotypes of Sotos syndrome and the functional domains of NSD1 protein were summarized by literature reviewing. Haploinsufficiency of the NSD1 gene is the main cause of Sotos syndrome.

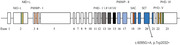

## 1 INTRODUCTION

Sotos syndrome (SS) (OMIM 11755), first described by Sotos et al. (1964) in 1964, is a growth disorder characterized by distinctive facial appearance, advanced bone age, macrocephaly, congenital visceral malformations, developmental delay, and epilepsy (Fortin et al., 2021; Höglund et al., 2003; Nagai et al., 2003; Ruhrman‐Shahar et al., 2022). The estimated incidence is 1 in 14000, which is one of the most common overgrowth syndromes (Lehman et al., 2012). Since 2002, it has been reported that up to 90% of patients are involved in alterations in the nuclear receptor binding SET domain protein 1 (*NSD1*) gene.

The nuclear receptor binding SET domain protein 1 (*NSD1*) gene (OMIM 606681) located on chromosome 5q35.3 consists of 23 exons, encoding nuclear receptor‐binding Su‐var, enhancer of zeste, and trithorax (SET) domain protein 1 with 2696 amino acids (Höglund et al., 2003). *NSD1* gene is expressed in the brain, spleen, thymus, kidney, skeletal muscle, and peripheral white blood cells (Kurotaki et al., 2002; Sohn et al., 2013). It has an 8088 bp open reading frame and the length of cDNA is 8552 bp (Kurotaki et al., 2001). NSD1 belonging to a family of nuclear receptors acts as a transcriptional regulator and histone methyltransferase by binding near various promoter elements to activate or repress transcription (Lucio‐Eterovic et al., 2010; Türkmen et al., 2003). The histone methyltransferase plays a role in catalyzing methylation of histone H3 lysine 36 (H3K36) and histone H4 lysine 20 (H4K20) (Oishi et al., 2020).

The nonsense mutation c.6095G > A (p.TRP2032*) in *NSD1* gene causing SS has not been reported before. It is concluded that this intragenic truncating variant was pathogenic and changed the protein structure.

## 2 METHODS

### 2.1 Trio‐based whole‐exome sequencing

Trio‐whole‐exome sequencing (WES) was performed for probands and her parents. Peripheral blood was extracted from all probands and their parents into EDTA anticoagulant tubes. The Agilent SureSelect was used to capture exons (Exome V6). After establishing the libraries, the data were sequenced on Illumina. Human Gene Mutations database (HGMD), Ingenuity online software system, and ClinVar database were employed to annotate related diseases. The candidate variations were verified by Sanger sequencing.

### 2.2 Literature review

The literature review was conducted by PubMed database using the terms (“Sotos syndrome” OR “Sotos’ Syndrome” OR “Soto Syndrome” OR “Soto's Syndrome” OR “Cerebral Gigantism”) AND “*NSD1*”. Publications from 1993 to 2023, including *NSD1* gene point mutations, ages from 0–18‐year old and detailed description of patient characteristics were selected.

## 3 RESULTS

### 3.1 Case details

The 3‐year‐and‐11‐month‐old female child is the second child of nonconsanguineous healthy parents and she has a healthy 10‐year‐old elder brother. She was born by full‐term cesarean section and her birth weight was 3300 g with a head circumference greater than the normal range. She had hand deformity manifested as flexing hands, and the fingers tilted outward could not be extended.

At 5 months, she was not able to look up or turn over. After rehabilitation training, she could walk with something and said simple words such as “Mom” and “dad.” After that, she stopped rehabilitation training. At the age of 3, she could walk and run alone, however, still could only speak some simple words. Currently, her height was in the 97th percentile of normal peers, and her weight was greater than the 97th percentile of normal children with the same age. She had the regression of language development and was unable to communicate routinely. She had a typical facial appearance, with protruding forehead, 57 cm of head circumference, oblique cleft eyes, widely spaced between eyes, long tip of mandible, and degeneration of hair on both temporal areas. Except for the thumb, the other four fingers of both hands were in the excessive outer booth, and the palms of both hands had no palm print.

The first seizure occurred when the child was 3‐and‐a‐half‐year old, and then repeated attacks, manifested as cyan lips, dyspnea, stiffness, accompanied by fever, which relieved itself after 2–3 min.

De novo heterozygous variant was found by WES in trios: *NSD1* (NM_022455.5), c.6095G > A, p.TRP2032*. This mutation was pathogenic in accordance with the American College of Medical Genetics and Genomics criteria (ACMG) guidelines.

**FIGURE 1:**
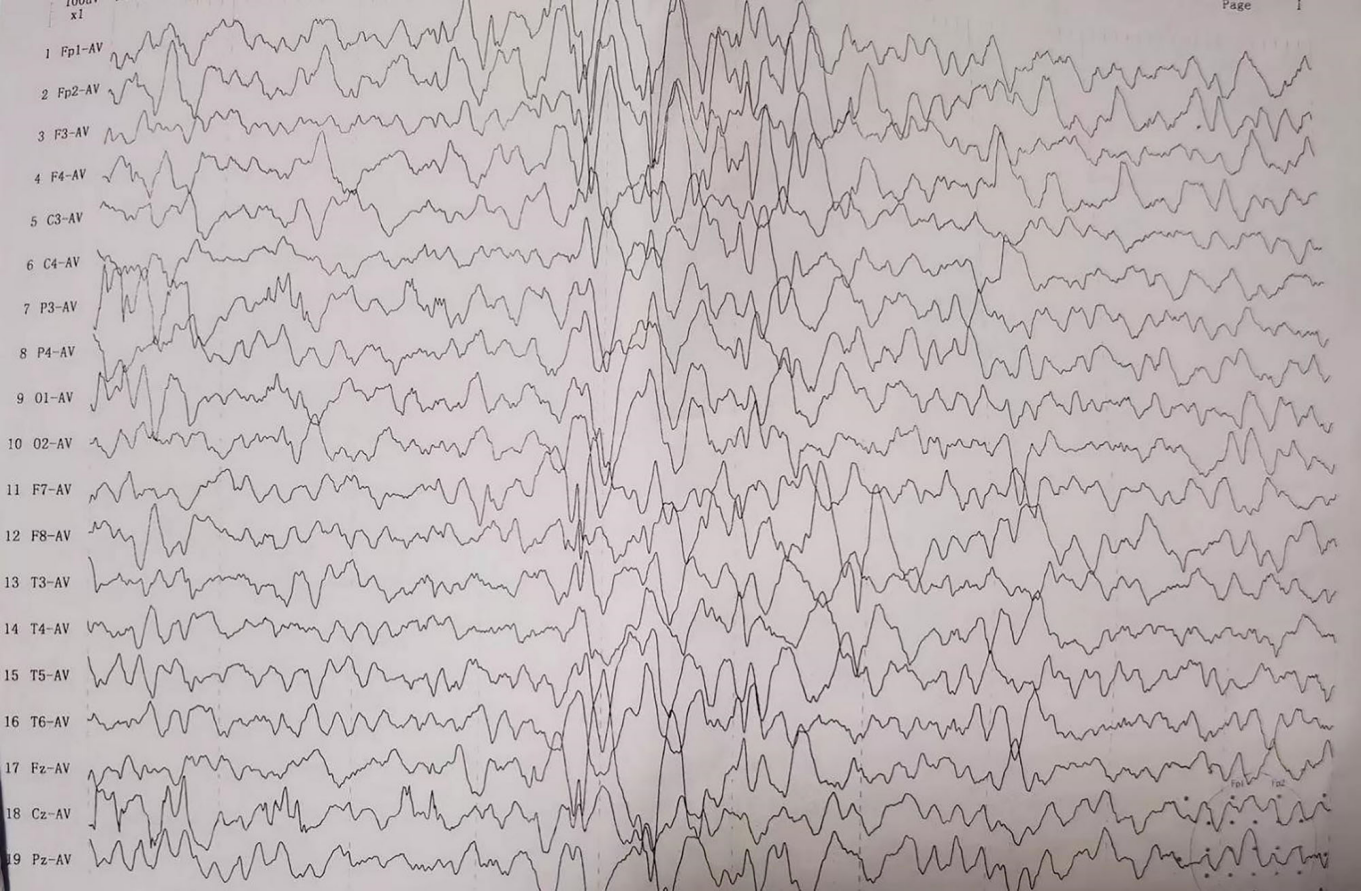
Electroencephalogram (EEG) test in the case with *NSD1* mutation. The results showed low frequencies of the background, multifocal slow‐spike, and sharp waves.

**FIGURE 2:**
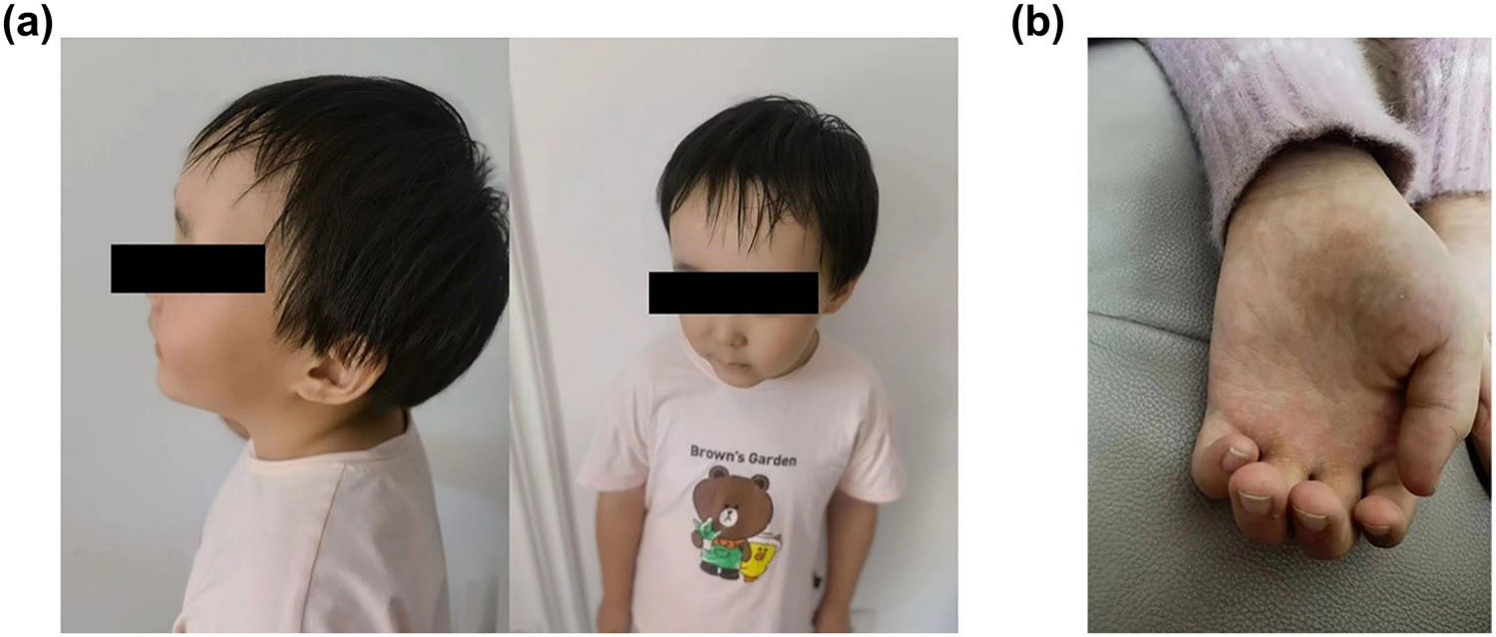
Typical facial appearance of the case. (A) Prominent forehead, high hairline, and frontotemporal sparse hairs. (B) For both hands, the fingers were in the excessive outer booth, and the palms had no palm print.

EEG showed abnormal discharges in occipital area (Figure 1). MRI displayed that the ventricle was slightly larger (Figure 2). According to the clinical manifestations of the patient, oxcarbazepine was given to control seizures. Rehabilitation training and regular follow‐up were required. The female child was diagnosed with SS on the basis of clinical manifestations, imaging, and genetic examination.

### 3.2 Literature review

We retrospected 12 articles and 14 children with SS caused by alterations of *NSD1* gene were included. The median age of the patients was 2‐and‐a‐half‐year old (range 8‐month to 10‐year old). The characteristics of 14 cases with SS were summarized through reviewing the literature shown in Table 1. The incidence of SS in male is higher than female (10:4). Most cases are sporadic, but some familial autosomal dominant cases have also been described (2/14) (Leventopoulos et al., 2009). Loss function of *NSD1* gene is due to microdeletion and mutations (missense variants, nonsense variants, splice variants, small insertions, and rearrangements) (Mencarelli et al., 2018), mainly leading to macrocephaly and overgrowth (12/14). Almost patients presented with development milestones delay in speech, dyskinesia, and different degrees of learning impairment (14/14) (Han et al., 2017; Piccione et al., 2011). Many cases were reported other clinical features, such as scoliosis, renal malformations, cardiac anomalies, syndactyly, neonatal hypotonia, joint hyperlaxity, reproductive system diseases, seizures, myopia, and conductive hearing loss (Foster et al., 2019; Sohn et al., 2013; Tatton‐Brown et al., 2005). Neuroimaging examinations commonly reported the ventricular enlargement, midline structure anomalies, and hypoplasia of the corpus callosum (Piccione et al., 2011). There is no correlation between genotype and phenotype, because cases with the same mutation sites usually have different clinical manifestations (Baujat & Cormier‐Daire, 2007).

**TABLE 1:** Characteristics of Sotos syndrome caused by alterations of *NSD1* gene.

Case report	Mutation sites	Protein variants	Mutation types	Origin	Age	Sex	Family history	Stature	Craniofacial features	Developmental delay or intellectual disabilities	Epilepsy	Imaging examination	EEG	Other organs damage
Panda et al. (2022)	c.3982A > T	p.Lys1328*	Nonsense	De novo	2 years old	Female	−	Advanced bone age	−	+	+	−	Frequent bilateral frontotemporal epileptiform discharges	−
Han et al. (2017)	c.2596G > T	p.Glu866*	Nonsense	De novo	5 ‐year old	Male	−	Advanced bone age	Triangular‐shaped face, prominent forehead, high hairline, downward‐slanting palpebral fissures, mild micrognathia, high arche palate, frontotemporal sparse hairs, and large ears	+	+	Ventriculomegaly, modest thinning of the corpus callosum and prominent extracereberal fluid‐filled spaces	Sharp‐and‐slow waves on the fronto‐central regions	Eyes and ears
Wejaphikul et al. (2015)	c.4710C > A	p.Cys1570*	Nonsense	De novo	3‐year‐8‐month old	Male	−	Normal stature	Prominent forehead and apparent hypertelorism, downward‐slanting palpebral fissures, a prominent jaw, micrognathia, and large ears	+	+	Periventricular leukomalacia with ventriculomegaly	Mild cerebral dysfunction	Hypoparathyroidism and kidney damage
Zhao (2018)	c.1177G > T	p.Glu386*	Nonsense	De novo	13‐month old	Male	−	Advanced bone age	Less hair on the top of the head, pointed mandible, distance between eyes	+	−	Ventricular enlargement	−	−
Zhao (2018)	c.1157T > G	p.Phe386Leu	Missense	De novo	10‐month old	Male	−	Advanced bone age	Frontal bossing, distance between eyes, high palatine arch	+	−	Ventricular enlargement	−	−
Mencarelli et al. (2018)	c.5867T > A	p.Leu1956Gln	Missense	De novo	4‐year old	Male	−	Advanced bone age	Trigonocephaly, frontal bossing, large ears, prominent chin, and high palate with dental malposition	+	−	NA	NA	Syndactyly
Su et al. (2011)	c.5990A > G	p.Tyr1997Cys	Missense	De novo	10‐year old	Female	−	Advanced bone age	Macrocephaly, prominent forehead, down‐slanting palpebral fissures, exotropia, amblyopia, and a pointed chin	+	−	Abnormal contour of the lateral ventricles bilaterally with squared‐off dilatation and symmetric, incomplete, thin septae in frontal horns bilaterally	NA	Agenesis of the left kidney
Lu et al. (2017)	c.5951G > A	p.Arg1984Gln	Missense	De novo	10‐year old	Female	−	Advanced bone age	NA	+	+	−	NA	Cleft lip and autism
Park et al. (2014)	c.6356delA	p.Asp2119Valfs*31	Deletion	Maternal	6‐month old	Female	Her mother also suffered from Sotos syndrome	Advanced bone age	Broad forehead, pointed chin, down‐slanted eyes, and high arched palate	+	−	Mild ventricular enlargement	NA	Right sided sensorineural hearing loss
Piccione et al. (2011)	Deletion of exon 14	NA	Deletion	De novo	4‐year old	Male	−	Advanced bone age	Macrocephaly, broad fore‐head, high hairline, narrow long face, large ears, pointed chin	+	−	Pellucidum cyst	NA	Reproductive system and heart
Höglund et al. (2003)	Deletion of a C at position 896 (896delC) in exon 2	NA	Deletion	Paternal	18‐month old	Male	His father also suffered from Sotos syndrome	Advanced bone age and overweight	Hypertelorism, prominent forehead, frontoparietal baldness straight palpebral fissures, small jaw was and high palate	+	−	Dilated lateral ventricles and wide frontal cortical space, and a thin corpus callosum	nonspecific abnormalities	Eyes, skin, and syndactyly
Verma et al. (2021)	c6076_6087del12	p.Asn2026_Thr2029del	Deletion	De novo	13‐month old	Male	−	Normal stature (large linear growth parameters for family history)	Triangular facies, frontal bossing, prominent occiput, and low set ears	+	−	Prominent subarachnoid spaces and a mild prominence of the lateral and third ventricles	NA	Prominence of the right rib cage with curvature of his spine
Özcabi et al. (2020)	c.4560dup	His1521Thrfs*9	Duplication	NA	9‐year old	Male	Consanguineous patients	Advanced bone age	Macro‐dolicocephaly, prominent forehead, and chin	+	−	Bilateral dilated ventricles, hydrocephalus, thin corpus callosum, pineal cyst	NA	Heart and eyes
Lu et al. (2017)	c.4809_4810insA	p.Arg1605LysfsTer13	Insertion	De novo	5‐year old	Male	−	Advanced bone age	Flat nasal bridge and an inverted triangular face	+	−	−	NA	−

Abbreviation: EEG, electroencephalogram.

## 4 DISCUSSION

In our case, the female patient had no family history, and the variant was de novo. This patient had typical facial abnormalities including a prominence of the forehead, frontotemporal hair sparsity, down‐slanting palpebral fissures, flushing, and a pointed chin similar to other reports (Grand et al., 2019; Sohn et al., 2013). In addition to the typical facial features of SS, she also showed hand deformities. Her height and weight were in the 97th percentile of normal peers, which is the representative feature of SSs. Growth velocity is higher than peers in their childhood, as adults, their height usually falls within the normal range (Özcabi et al., 2020). The girl did not perform organs damage like congenital heart disease, kidney repair. But she had developmental delay and febrile seizure. SS is an overgrowth disease, but many patients also suffer from epilepsy. The seizures are usually self‐limited. Staring spells were the most frequent seizure type, followed by febrile seizures and afebrile bilateral tonic–clonic seizures (Fortin et al., 2021). SS also can be complicated by mental and psychological problems, such as anxiety, depression, attention‐deficit hyperactivity disorder, and autism spectrum disorder (Özcabi et al., 2020). These symptoms were absence in our case.

The main function of *NSD1* gene is transcriptional activation and repression through chromatin modification. It is a histone methyltransferase that primarily dimethylates Lys‐36 of histone H3 (H3K36me2), trimethylates Lys‐20 of histone H4 (H4K20me3) as well (Watanabe et al., 2020). The methylation of H3K36 marks the expressed genes and is related to the inhibition of the initiation of intragenic transcription in the expressed genes (Lee & Shilatifard, 2007; Pasillas et al., 2011). H4K20 methylation participated in serious activities, including mitosis, gene activation and repression, chromatin condensation, and DNA‐damage checkpoint signaling (Rayasam et al., 2003). Another function of *NSD1* gene is acting as a co‐inhibitor of growth promoting genes, which will have a positive or negative impact on the transcription of nuclear receptors (e.g., estrogens, retinoic acid, and thymoid hormone receptors) depending on the cellular environment (Ha et al., 2016). In many cases, haploinsufficiency of *NSD1* like nonsense or missense mutations and deletions was suggested to be associated with overgrowth in stature and face. Thus, the main cause of SS is the haploinsufficiency of *NSD1* gene in the distal long arm of chromosome 5 (5q35.2–q35.3) (Kurotaki et al., 2002). But in some cases, the 5q35 microduplication was confirmed and the patients manifest “reversed SS” as short stature and microcephaly without facial features (Franco et al., 2010; Reis et al., 2017).

**FIGURE 3:**
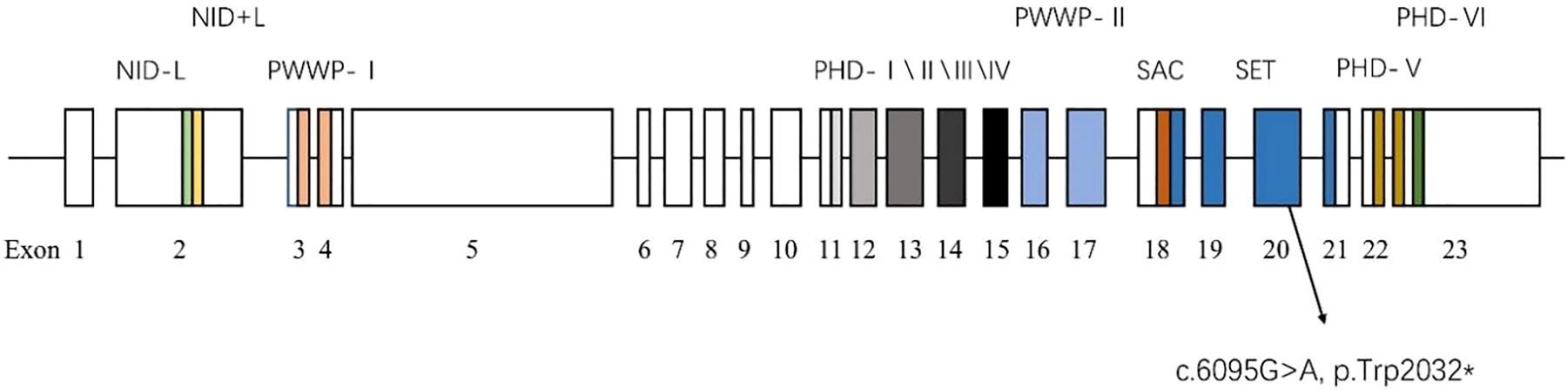
Schematic representation of the functional domains in NSD1 protein. Distinct colored boxes represent different domains. The 23 boxes present with exons and introns are shown as lines with the exon numbers underneath and the domain names above. The location of the novel nonsense mutation is pointed out.

The NSD1 contains multiple functional domains, including two nuclear receptor interaction domains (NID − L and NID + L), Pro‐Tryptophan‐Proline (PWWP) I and II, six plant homeo domains (PHD), SET‐associated Cys‐rich (SAC), SET, post‐SET, and a PHD fingerlike Cys‐His rich domain (Berardi et al., 2016; Tatton‐Brown & Rahman, 2004; Türkmen et al., 2003). The main functional domains have been shown in Figure 3. NID − L and NID+L are corepressors and coactivators, respectively. Two different nuclear receptor interaction domains permit *NSD1* to negatively and positively regulate transcription (Tatton‐Brown et al., 2005). SET is a highly conserved catalytic domain, which is the most important area of *NSD1*. Other domains, such as PWWP and PHD, are auxiliary (Qiao et al., 2011). It suggested that PWWP domains are involved in protein–protein interactions, they act as the recognition sites of other proteins on NSD1, thereby activating methyltransferase activity (Watanabe et al., 2020). PHD finger domains have effects on chromatin mediated transcriptional regulation (Türkmen et al., 2003; Verma et al., 2021). SAC domain may play a part in chromosome binding (Türkmen et al., 2003).

The heterozygous mutation c.6095G > A (p.Trp2032*) occurred on exon 20 of *NSD1* gene and was located in the SET domain. It has never been described before and cannot be searched in various databases (e.g., Exome Variant Server, clinivar, HGMD, and gnomAD). According to the ACMG, the c.6095G > A (p.Trp2032*) variant can be classified as likely pathogenic. The nonsense variant led to the codon TGG encoding tryptophan at position 2032 changed into the stop codon TGA, and the early appearance of the stop codon at amino acid 2032. As a result, large fragments of NSD1 protein were truncated.

In this case report, the nonsense mutation attacks in SET domain which is the core during methylation. SET can catalyze the transfer of methyl groups to lysine residues at the tail of histones (Watanabe et al., 2020). Due to the early appearance of the termination codon, the truncated protein lost post SET loop and two PHD fingers. Post SET loop is essential for the methyltransferase activity of NSD with nucleosome as substrate. A post‐SET extension is responsible for nucleosome binding (Allali‐Hassani et al., 2014; Graham et al., 2016). Berardi et al. (2016) indicated that PHD V combining with PHD VI can interact with the C2HR region of Nizp1, a transcription inhibitor. Nonsense mutations are able to make the truncated protein to be unstable and swift degradation after synthesis, that is, nonsense mediated degradation (NMD). We have mentioned above that haploinsufficiency of NSD1 is the major cause of SS. The nonsense mutation we reported was likely to have triggered the NMD mechanism, leading to rapid degradation of the faulty RNA, avoiding truncated proteins in large quantities (Chang et al., 2007; Holbrook et al., 2004; Malan et al., 2010). We deduced that these factors mentioned above account for the pathogenicity of the c.6095G > A (p.Trp2032*) variant.

## 5 CONCLUSION

We report a female child with SS carrying a de novo nonsense mutation in *NSD1* gene. The phenotypes of SS and the function of NSD1 protein and each domain were summarized by literature reviewing. We hypothesize the mechanism of this intragenic truncating mutation affecting protein function.

## AUTHOR CONTRIBUTIONS

All the mentioned authors have participated in the report. Xinting Liu and Gang Zhu reviewed the literature and wrote the first draft of the manuscript. Guang Yang and Jing Wang supervised the article. All authors read, revised, and approved the final version of the manuscript.

## CONFLICT OF INTEREST STATEMENT

The authors declare no conflicts of interest.

### PEER REVIEW

The peer review history for this article is available at https://publons.com/publon/10.1002/brb3.3290.

## Data Availability

Data sharing is not applicable to this article because no other data were analyzed in this study.

## References

[brb33290-bib-0001] Allali‐Hassani, A. , Kuznetsova, E. , Hajian, T. , Wu, H. , Dombrovski, L. , Li, Y. , Gräslund, S. , Arrowsmith, C. H. , Schapira, M. , & Vedadi, M. (2014). A basic post‐SET extension of NSDs is essential for nucleosome binding in vitro. Journal of Biomolecular Screening, 19(6), 928–935. 10.1177/1087057114525854 24595546

[brb33290-bib-0002] Baujat, G. , & Cormier‐Daire, V. (2007). Sotos syndrome. Orphanet Journal of Rare Diseases, 2, 36. 10.1186/1750-1172-2-36 17825104 PMC2018686

[brb33290-bib-0003] Berardi, A. , Quilici, G. , Spiliotopoulos, D. , Corral‐Rodriguez, M. A. , Martin‐Garcia, F. , Degano, M. , Tonon, G. , Ghitti, M. , & Musco, G. (2016). Structural basis for PHDVC5HCHNSD1–C2HRNizp1 interaction: Implications for Sotos syndrome. Nucleic Acids Research, 44(7), 3448–3463. 10.1093/nar/gkw103 26896805 PMC4838375

[brb33290-bib-0042] Chang, Y. F. , Imam, J. S. , & Wilkinson, M. F. (2007). The nonsense‐mediated decay RNA surveillance pathway. Annual Review of Biochemistry, 76, 51–74. 10.1146/annurev.biochem.76.050106.093909 17352659

[brb33290-bib-0004] Fortin, O. , Vincelette, C. , Khan, A. Q. , Berrahmoune, S. , Dassi, C. , Karimi, M. , Scheffer, I. E. , Lu, J. , Davis, K. , & Myers, K. A. (2021). Seizures in Sotos syndrome: Phenotyping in 49 patients. Epilepsia Open, 6(2), 425–430. 10.1002/epi4.12484 34033256 PMC8166795

[brb33290-bib-0005] Foster, A. , Zachariou, A. , Loveday, C. , Ashraf, T. , Blair, E. , Clayton‐Smith, J. , Dorkins, H. , Fryer, A. , Gener, B. , Goudie, D. , Henderson, A. , Irving, M. , Joss, S. , Keeley, V. , Lahiri, N. , Lynch, S. A. , Mansour, S. , McCann, E. , Morton, J. , … Tatton‐Brown, K. (2019). The phenotype of Sotos syndrome in adulthood: A review of 44 individuals. American Journal of Medical Genetics Part C, Seminars in Medical Genetics, 181(4), 502–508. 10.1002/ajmg.c.31738 31479583

[brb33290-bib-0006] Franco, L. M. , de Ravel, T. , Graham, B. H. , Frenkel, S. M. , Van Driessche, J. , Stankiewicz, P. , Lupski, J. R. , Vermeesch, J. R. , & Cheung, S. W. (2010). A syndrome of short stature, microcephaly and speech delay is associated with duplications reciprocal to the common Sotos syndrome deletion. European Journal of Human Genetics, 18(2), 258–261. 10.1038/ejhg.2009.164 19844260 PMC2987195

[brb33290-bib-0007] Graham, S. E. , Tweedy, S. E. , & Carlson, H. A. (2016). Dynamic behavior of the post‐SET loop region of NSD1: Implications for histone binding and drug development. Protein Science, 25(5), 1021–1029. 10.1002/pro.2912 26940890 PMC4838653

[brb33290-bib-0008] Grand, K. , Gonzalez‐Gandolfi, C. , Ackermann, A. M. , Aljeaid, D. , Bedoukian, E. , Bird, L. M. , de Leon, D. D. , Diaz, J. , Hopkin, R. J. , Kadakia, S. P. , Keena, B. , Klein, K. O. , Krantz, I. , Leon, E. , Lord, K. , McDougall, C. , Medne, L. , Skraban, C. M. , Stanley, C. A. , … Kalish, J. M. (2019). Hyperinsulinemic hypoglycemia in seven patients with de novo NSD1 mutations. American Journal of Medical Genetics. Part A, 179(4), 542–551. 10.1002/ajmg.a.61062 30719864 PMC6454923

[brb33290-bib-0009] Ha, K. , Anand, P. , Lee, J. A. , Jones, J. R. , Kim, C. A. , Bertola, D. R. , Labonne, J. D. J. , Layman, L. C. , Wenzel, W. , & Kim, H. G. (2016). Steric clash in the SET domain of histone methyltransferase NSD1 as a cause of Sotos syndrome and its genetic heterogeneity in a Brazilian cohort. Genes (Basel), 7(11), 96. 10.3390/genes7110096 27834868 PMC5126782

[brb33290-bib-0010] Han, J. Y. , Lee, I. G. , Jang, W. , Shin, S. , Park, J. , & Kim, M. (2017). Identification of a novel de novo nonsense mutation of the NSD1 gene in monozygotic twins discordant for Sotos syndrome. Clinica Chimica Acta, 470, 31–35. 10.1016/j.cca.2017.04.025 28457852

[brb33290-bib-0011] Höglund, P. , Kurotaki, N. , Kytölä, S. , Miyake, N. , Somer, M. , & Matsumoto, N. (2003). Familial Sotos syndrome is caused by a novel 1 bp deletion of the NSD1 gene. Journal of Medical Genetics, 40(1), 51–54. 10.1136/jmg.40.1.51 12525543 PMC1735268

[brb33290-bib-0043] Holbrook, J. A. , Neu‐Yilik, G. , Hentze, M. W. , & Kulozik, A. E. (2004). Nonsense‐mediated decay approaches the clinic. Nature Genetics, 36(8), 801–808. 10.1038/ng1403 15284851

[brb33290-bib-0012] Kurotaki, N. , Harada, N. , Yoshiura, K. , Sugano, S. , Niikawa, N. , & Matsumoto, N. (2001). Molecular characterization of NSD1, a human homologue of the mouse Nsd1 gene. Gene, 279(2), 197–204. 10.1016/S0378-1119(01)00750-8 11733144

[brb33290-bib-0013] Kurotaki, N. , Imaizumi, K. , Harada, N. , Masuno, M. , Kondoh, T. , Nagai, T. , Ohashi, H. , Naritomi, K. , Tsukahara, M. , Makita, Y. , Sugimoto, T. , Sonoda, T. , Hasegawa, T. , Chinen, Y. , Ha, H.‐A. T. , Kinoshita, A. , Mizuguchi, T. , Ki, K.‐I. Y. , Ohta, T. , … Matsumoto, N. (2002). Haploinsufficiency of NSD1 causes Sotos syndrome. Nature Genetics, 30(4), 365–366. 10.1038/ng863 11896389

[brb33290-bib-0014] Lee, J. S. , & Shilatifard, A. (2007). A site to remember: H3K36 methylation a mark for histone deacetylation. Mutation Research, 618(1–2), 130–134. 10.1016/j.mrfmmm.2006.08.014 17346757

[brb33290-bib-0015] Lehman, A. M. , du Souich, C. , Chai, D. , Eydoux, P. , Huang, J. L. , Fok, A. K. , Avila, L. , Swingland, J. , Delaney, A. D. , McGillivray, B. , Goldowitz, D. , Argiropoulos, B. , Kobor, M. S. , & Boerkoel, C. F. (2012). 19p13.2 microduplication causes a Sotos syndrome‐like phenotype and alters gene expression. Clinical Genetics, 81(1), 56–63. 10.1111/j.1399-0004.2010.01615.x 21204797

[brb33290-bib-0016] Leventopoulos, G. , Kitsiou‐Tzeli, S. , Kritikos, K. , Psoni, S. , Mavrou, A. , Kanavakis, E. , & Fryssira, H. (2009). A clinical study of Sotos syndrome patients with review of the literature. Pediatric Neurology, 40(5), 357–364. 10.1016/j.pediatrneurol.2008.11.013 19380072

[brb33290-bib-0017] Lu, Y. , Chong, P. F. , Kira, R. , Seto, T. , Ondo, Y. , Shimojima, K. , & Yamamoto, T. (2017). Mutations in NSD1 and NFIX in three patients with clinical features of Sotos syndrome and Malan syndrome. Journal of Pediatric Genetics, 6(4), 234–237. 10.1055/s-0037-1603194 29142766 PMC5683957

[brb33290-bib-0018] Lucio‐Eterovic, A. K. , Singh, M. M. , Gardner, J. E. , Veerappan, C. S. , Rice, J. C. , & Carpenter, P. B. (2010). Role for the nuclear receptor‐binding SET domain protein 1 (NSD1) methyltransferase in coordinating lysine 36 methylation at histone 3 with RNA polymerase II function. Proceedings National Academy of Science USA, 107(39), 16952–16957. 10.1073/pnas.1002653107 PMC2947892 20837538

[brb33290-bib-0044] Malan, V. , Rajan, D. , Thomas, S. , Shaw, A. C. , Louis Dit Picard, H. , Layet, V. , Till, M. , van Haeringen, A. , Mortier, G. , Nampoothiri, S. , Puseljić, S. , Legeai‐Mallet, L. , Carter, N. P. , Vekemans, M. , Munnich, A. , Hennekam, R. C. , Colleaux, L. , & Cormier‐Daire, V. (2010). Distinct effects of allelic NFIX mutations on nonsense‐mediated mRNA decay engender either a Sotos‐like or a Marshall‐Smith syndrome. American Journal of Human Genetics, 87(2), 189–198. 10.1016/j.ajhg.2010.07.001 20673863 PMC2917711

[brb33290-bib-0019] Mencarelli, A. , Prontera, P. , Mencarelli, A. , Rogaia, D. , Stangoni, G. , Cecconi, M. , & Esposito, S. (2018). Expanding the clinical spectrum of Sotos syndrome in a patient with the new “c.[5867T>A]+[ = ]”; “p.[Leu1956Gln]+[ = ]” NSD1 missense mutation and complex skin hamartoma. International Journal of Molecular Sciences, 19(10), 3189. 10.3390/ijms19103189 30332768 PMC6213993

[brb33290-bib-0020] Nagai, T. , Matsumoto, N. , Kurotaki, N. , Harada, N. , Niikawa, N. , Ogata, T. , Imaizumi, K. , Kurosawa, K. , Kondoh, T. , Ohashi, H. , Tsukahara, M. , Makita, Y. , Sugimoto, T. , Sonoda, T. , Yokoyama, T. , Uetake, K. , Sakazume, S. , Fukushima, Y. , & Naritomi, K. (2003). Sotos syndrome and haploinsufficiency of NSD1: Clinical features of intragenic mutations and submicroscopic deletions. Journal of Medical Genetics, 40(4), 285–289. 10.1136/jmg.40.4.285 12676901 PMC1735419

[brb33290-bib-0021] Oishi, S. , Zalucki, O. , Vega, M. S. , Harkins, D. , Harvey, T. J. , Kasherman, M. , Davila, R. A. , Hale, L. , White, M. , Piltz, S. , Thomas, P. , Burne, T. H. J. , Harris, L. , Piper, M. , & Piper, M. (2020). Investigating cortical features of Sotos syndrome using mice heterozygous for Nsd1. Genes, Brain, and Behavior, 19(4), e12637. 10.1111/gbb.12637 31909872

[brb33290-bib-0022] Özcabi, B. , Akay, G. , Yesil, G. , Uyur Yalcin, E. , & Kirmizibekmez, H. (2020). A case of Sotos syndrome caused by a novel variant in the NSD1 gene: A proposed rationale to treat accompanying precocious puberty. Acta Endocrinol (Buchar), 16(2), 245–249. 10.4183/aeb.2020.245 33029244 PMC7535889

[brb33290-bib-0023] Panda, P. K. , Nandani, R. , Mehta, S. , & Sharawat, I. K. (2022). Developmental delay and epilepsy without Gigantism: An unusual presentation of Soto's syndrome due to a novel mutation in the NSD1 gene. Annals of Indian Academy of Neurology, 25(1), 152–153. 10.4103/aian.AIAN_209_21 35342273 PMC8954316

[brb33290-bib-0024] Park, S. H. , Lee, J. E. , Sohn, Y. B. , & Ko, J. M. (2014). First identified Korean family with Sotos syndrome caused by a novel intragenic mutation in NSD1. Annals of Clinical and Laboratory Science, 44(2), 228–231. 24795065

[brb33290-bib-0025] Pasillas, M. P. , Shah, M. , & Kamps, M. P. (2011). NSD1 PHD domains bind methylated H3K4 and H3K9 using interactions disrupted by point mutations in human Sotos syndrome. Human Mutation, 32(3), 292–298. 10.1002/humu.21424 21972110

[brb33290-bib-0026] Piccione, M. , Consiglio, V. , Di Fiore, A. , Grasso, M. , Cecconi, M. , Perroni, L. , & Corsello, G. (2011). Deletion of NSD1 exon 14 in Sotos syndrome: First description. Journal of Genetics, 90(1), 119–123. 10.1007/s12041-011-0017-6 21677396

[brb33290-bib-0027] Qiao, Q. , Li, Y. , Chen, Z. , Wang, M. , Reinberg, D. , & Xu, R. M. (2011). The structure of NSD1 reveals an autoregulatory mechanism underlying histone H3K36 methylation. Journal of Biological Chemistry, 286(10), 8361–8368. 10.1074/jbc.M110.204115 21196496 PMC3048720

[brb33290-bib-0028] Rayasam, G. V. , Wendling, O. , Angrand, P. O. , Mark, M. , Niederreither, K. , Song, L. , Lerouge, T. , Hager, G. L. , Chambon, P. , & Losson, R. (2003). NSD1 is essential for early post‐implantation development and has a catalytically active SET domain. Embo Journal, 22(12), 3153–3163. 10.1093/emboj/cdg288 12805229 PMC162140

[brb33290-bib-0029] Reis, F. G. , Pinto, I. P. , Minasi, L. B. , Melo, A. V. , Cunha, D. M. , Ribeiro, C. L. , da Silva, C. C. , de M Silva, D. , & da Cruz, A. D. (2017). A rare case of a boy with de novo microduplication at 5q35.2q35.3 from central Brazil. Genetics and Molecular Research [Electronic Resource], 16(1), 10.4238/gmr16019197 28128410

[brb33290-bib-0030] Ruhrman‐Shahar, N. , Assia Batzir, N. , Lidzbarsky, G. A. , Bazak, L. , Magal, N. , & Basel‐Salmon, L. (2022). A nonsense variant in the second exon of the canonical transcript of NSD1 does not cause Sotos syndrome. American Journal of Medical Genetics. Part A, 188(1), 369–372. 10.1002/ajmg.a.62519 34559457

[brb33290-bib-0031] Sohn, Y. B. , Lee, C. G. , Ko, J. M. , Yang, J. A. , Yun, J. N. , Jung, E. J. , Jin, H.‐S. , Park, S.‐J. , & Jeong, S. Y. (2013). Clinical and genetic spectrum of 18 unrelated Korean patients with Sotos syndrome: Frequent 5q35 microdeletion and identification of four novel NSD1 mutations. Journal of Human Genetics, 58(2), 73–77. 10.1038/jhg.2012.135 23190751

[brb33290-bib-0032] Sotos, J. F. , Dodge, P. R. , Muirhead, D. , Crawford, J. D. , & Talbot, N. B. (1964). Cerebral gigantism in childhood. A syndrome of excessively rapid growth and acromegalic features and a nonprogressive neurologic disorder. New England Journal of Medicine, 271, 109–116. 10.1056/NEJM196407162710301 14148233

[brb33290-bib-0033] Su, P. H. , Yu, J. S. , Chen, S. J. , Chen, J. Y. , & Tsao, T. F. (2011). Persistent falcine sinus and unilateral renal agenesis in a girl with Sotos syndrome. Clinical Dysmorphology, 20(1), 42–46. 10.1097/MCD.0b013e32833ff281 21084978

[brb33290-bib-0034] Tatton‐Brown, K. , Douglas, J. , Coleman, K. , Baujat, G. , Cole, T. R. , Das, S. , Horn, D. , Hughes, H. E. , Temple, I. K. , Faravelli, F. , Waggoner, D. , Turkmen, S. , Cormier‐Daire, V. , Irrthum, A. , & Rahman, N. , Childhood Overgrowth Collaboration . (2005). Genotype‐phenotype associations in Sotos syndrome: An analysis of 266 individuals with NSD1 aberrations. American Journal of Human Genetics, 77(2), 193–204. 10.1086/432082 15942875 PMC1224542

[brb33290-bib-0035] Tatton‐Brown, K. , & Rahman, N. (2004). Clinical features of NSD1‐positive Sotos syndrome. Clinical Dysmorphology, 13(4), 199–204. 10.1097/00019605-200410000-00001 15365454

[brb33290-bib-0036] Türkmen, S. , Gillessen‐Kaesbach, G. , Meinecke, P. , Albrecht, B. , Neumann, L. M. , Hesse, V. , Palanduz, S. , Balg, S. , Majewski, F. , Fuchs, S. , Zschieschang, P. , Greiwe, M. , Mennicke, K. , Kreuz, F. R. , Dehmel, H. J. , Rodeck, B. , Kunze, J. , Tinschert, S. , Mundlos, S. , … Horn, D. (2003). Mutations in NSD1 are responsible for Sotos syndrome, but are not a frequent finding in other overgrowth phenotypes. European Journal of Human Genetics, 11(11), 858–865. 10.1038/sj.ejhg.5201050 14571271

[brb33290-bib-0037] Verma, A. , Salehi, P. , Hing, A. , & Roberts, A. J. C. (2021). Sotos syndrome with a novel mutation in the NSD1 gene associated with congenital hypothyroidism. International Journal of Pediatrics and Adolescent Medicine, 8(3), 191–194. 10.1016/j.ijpam.2020.06.001 34350334 PMC8319649

[brb33290-bib-0038] Watanabe, H. , Higashimoto, K. , Miyake, N. , Morita, S. , Horii, T. , Kimura, M. , Suzuki, T. , Maeda, T. , Hidaka, H. , Aoki, S. , Yatsuki, H. , Okamoto, N. , Uemura, T. , Hatada, I. , Matsumoto, N. , Soejima, H. , & Soejima, H. (2020). DNA methylation analysis of multiple imprinted DMRs in Sotos syndrome reveals IGF2‐DMR0 as a DNA methylation‐dependent, P0 promoter‐specific enhancer. Faseb Journal, 34(1), 960–973. 10.1096/fj.201901757R 31914674 PMC6973060

[brb33290-bib-0039] Wejaphikul, K. , Cho, S. Y. , Huh, R. , Kwun, Y. , Lee, J. , Ki, C. S. , & Jin, D. K. (2015). Hypoparathyroidism in a 3‐year‐old Korean boy with Sotos syndrome and a novel mutation in NSD1. Annals of Clinical and Laboratory Science, 45(2), 215–218. 25887879

[brb33290-bib-0040] Zhao, M. (2018). Clinical phenotypes and a genetic analysis of patients with Sotos syndrome. Zhongguo Dang Dai Er Ke Za Zhi, 20(6), 481–484. 10.7499/j.issn.1008-8830.2018.06.010 29972123 PMC7389955

